# Cancer as a novel risk factor for major cardiovascular adverse events in secondary prevention

**DOI:** 10.1016/j.ijcrp.2025.200501

**Published:** 2025-08-26

**Authors:** Renzo Melchiori, Sara Diaz Saravia, Pablo M. Rubio, Lucas Szlaien, Romina Mouriño, Martin O'Flaherty, Manglio Rizzo, Alejandro Hita

**Affiliations:** a Department of Cardiology and Echocardiography, Hospital Universitario Austral, Austral University, Derqui-Pilar, Buenos Aires, Argentina; b Department of Medicine, Icahn School of Medicine at Mount Sinai, New York, NY, USA; c Interventional Cardiology, Medstar Washington Hospital Center, Washington, D.C, USA; d Department of Cardiology, Hospital Posadas, Buenos Aires, Argentina; e Department of Medicine, Loyola University Medical Center, Chicago, USA; f Department of Public Health, Policy and Systems, University of Liverpool, Liverpool, United Kingdom; g Cancer Immunobiology Laboratory, Instituto de Investigaciones en Medicina Traslacional, Universidad Austral-Consejo Nacional de Investigaciones Cientificas y Tecnologicas (CONICET), Buenos Aires, Argentina; h Department of Clinical Oncology, Hospital Universitario Austral, Derqui-Pilar, Buenos Aires, Argentina

**Keywords:** Secondary prevention, Coronary artery disease, Cancer, Risk factor

## Abstract

**Introduction:**

The inflammatory mechanisms of cancer can be associated with atherosclerosis development and progression. Although the incidence of events in secondary prevention following a first acute coronary syndrome is poorly documented.

**Methods:**

A retrospective cohort study including patients who underwent a coronary angiography for first Acute Coronary Syndrome (ACS), and without prior history of Major Cardiovascular Events (MACE) from 2008 to 2023 was analyzed. Included patients were grouped according to the absence or presence of cancer: G1 non-oncologic, and G2 oncologic (either prior or current history). We compared the incidence rate ratio of MACE within 3 years after ACS between groups Time-to-event analysis was conducted through proportional Cox regression analysis, estimating hazard ratio, and corresponding 95 % confidence intervals (95 % CI)

**Results:**

Of 937 patients who underwent a coronary angiography, 787 patients were included, of which 88.7 % (n = 698) presented without cancer. Over a median follow-up time of 48 months [IQR = 14–72], the incidence rate of MACE was 4.4 cases per 1000 patients/months of follow-up (n = 173 MACE events). When comparing both groups, the incidence rate ratio of MACE was 1.9 (95 % CI 1.24–2.99), significantly increased in the cancer group (P = 0.0032). Cancer was an independent predictor of MACE after adjustment for traditional cardiovascular risk factors (HR 1.84, 95 % CI 1.19–2.85; P = 0.006).

**Conclusions:**

Patients with cancer represent a novel independent risk factor for MACE, even following secondary preventive therapies. These results highlight future endpoints for cardiovascular prevention and further public health interventions in this population.

## Graphical abstract

**Figure:**
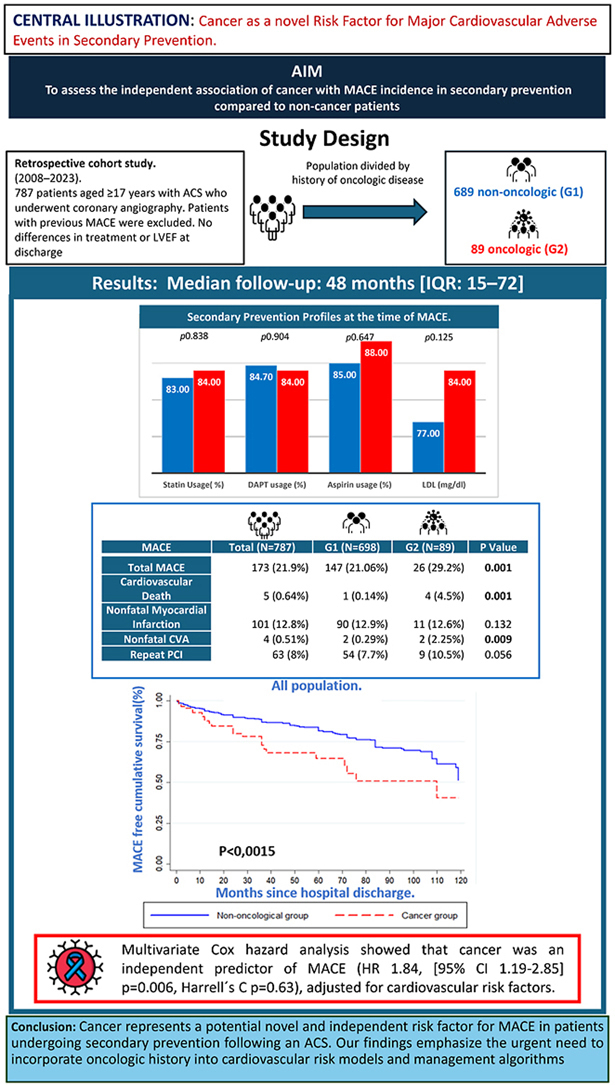

## 1 Introduction

Cancer is the second cause of death worldwide, only surpassed by cardiovascular diseases [1]. In recent decades, cancer mortality rates have decreased, with a consequent increase in overall survival [2]. For those patients who survive cancer, oncological treatments and cardiovascular and cerebrovascular diseases become the main causes of mortality [3,4]. In 2022, there were approximately 4.2 million cases of cancer in the region of the Americas, and this number is projected to increase up to 6.7 million in 2045 according to the Pan-American Health Organization. This growing number calls for a better understanding of the relationship of cancer and cardiovascular disease in the long term.

Multiple studies have emphasized the relationship between the pathophysiological mechanisms of cancer and cardiovascular disease [5,6]. There is emerging evidence on the role of oncological disease as a precipitator of chronic inflammation and long-term endothelial damage [[7], [8], [9], [10], [11], [12]]. Although there are multiple registries and publications related to the increased cardiovascular events in the cancer population [3,5,[13], [14], [15], [16], [17]], which have generated specific recommendations for primary prevention in this patient group [18,19], the evidence for secondary prevention is scarce.

However, less is known in terms of the excess risk of cancer on Major Adverse Cardiovascular Events (MACE) in the context of secondary prevention. If this association exists, its role in modifying management and prognostic strategies will need to be defined, implying a significant impact in public health and cardiovascular prevention. Therefore, we sought to determine the independent association of oncological disease and its effect on the incidence of MACE compared to patients without cancer in the secondary prevention setting.

## 2 Methods

### 2.1 Setting and study design

A retrospective cohort study was conducted including adult patients (>17 years of age) who were admitted to the Cardiac Care Unit at the Austral University Hospital with Acute Coronary Syndrome (ACS) from January 1st^,^ 2008 to March 31st^,^ 2023. This registry was formed through the review of Electronic Medical Records of patients presenting with ACS during that period defined by an elevated troponin level plus at least one of the following:

• Symptoms of acute myocardial ischemia
• New ischemic changes in the ECG
• Development of pathological Q waves
• Imaging evidence of loss of viable myocardium or new regional wall motion abnormalities consistent with an ischemic pattern

Subjects were selected according to the eligibility criteria defined (see section “Eligibility Criteria"). Institutional Ethical Approval was granted by the Ethics Board and written informed consent was obtained from the patients before their admission to the institution (Institutional Evaluation Committee of the Universidad Austral, CIE No. P21-022).

### 2.2 Eligibility criteria

Patients eligible for this study included those who presented with positive cardiac biomarkers along with at least one of the following criteria: electrocardiographic changes, anginal symptoms, and/or new regional wall motion abnormalities, and who underwent Left Heart Catheterization (LHC) as part of the diagnostic and therapeutic strategy during their first hospitalization for Acute Coronary Syndrome (ACS).

Patients with a prior history of Myocardial Infarction (MI), Cerebrovascular Accident (CVA), Percutaneous Coronary Intervention (PCI), or Coronary Artery Bypass Grafting (CABG) were excluded.

### 2.3 Exposure variable of interest

The exposure group was defined as any active oncological history (under treatment) or referred, regardless of its duration, prior to entry into the cohort, irrespective of the type of treatment received. The cancer history necessary for our study was obtained from the patient's electronic medical records. It was characterized by the type of neoplasm, defining hematologic neoplasia as leukemia, lymphomas, myeloproliferative syndromes, and multiple myeloma, and solid organ tumors as any tumor that is non-hematological, establishing cellular type where possible. The years since diagnosis were determined as the time from cancer diagnosis to admission to the coronary unit or cardiovascular study as part of the admission. The oncological stage was established according to the TNM classification system.

Regarding cancer therapies, it was assessed whether patients were under active treatment or not. Active cancer was defined as any patient undergoing oncological treatment who receives specific therapy for it within the 90 days prior to admission to the ACS and/or is currently in a restaging plan. Active chemotherapy was defined as having received chemotherapy or immunotherapy within 60 days prior to initial hospital presentation. Specific treatment types were categorized into chemotherapy, immunotherapy, hormone therapy, and radiotherapy. Radiation therapy history was defined as radiation treatment to any organ, establishing the cumulative dose, if possible, as the oncological treatment may have been administered at another institution. It was also determined whether anthracyclines were used in oncological treatment. The time since completion of treatment was established in years at the time of admission for acute coronary syndrome (ACS). Skin cancer basal cell carcinoma or squamous cell carcinoma were excluded.

### 2.4 Other exposure variables of interest and co-interventions

The known risk factors were collected by the attending physician upon admission for the ACS in the electronic medical records, including hypertension defined as systolic blood pressure greater than or equal to 140 mmHg and/or diastolic blood pressure greater than or equal to 90 mmHg or the use of antihypertensive medications at the time of hospitalization. Diabetes mellitus was defined as HbA1c levels greater than 6.5 % in the laboratory and/or documented history in the medical record and/or use of any glucose-lowering medication, either oral agents or insulin.

Dyslipidemia was defined as for total cholesterol >200 mg7/dl, LDL cholesterol >130, and/or triglycerides >150, as well as the use of lipid-lowering medications.

Sedentary lifestyle was considered a dichotomous variable for any patient who, during the admission interview to the coronary care unit, reported engaging in less than 150 min of physical activity per week. No levels of physical activity were assigned. Obesity was defined as a body mass index equal to or greater than 30 and family history included the presence of myocardial infarction or stroke in a first-degree relative before the age of 55 for men or 65 for women.

The clinical variables and laboratory tests to assess adherence to secondary prevention goals were rate of statins rate, dosing of statins (standardized to equivalent doses of atorvastatin), LDL value (mg/dl), use of dual antiplatelet therapy (DAPT), duration of DAPT and use of Aspirin. These parameters were measured in both oncology and non-oncology patients as part of their follow-up.

### 2.5 Primary outcome assessment, definition, and statistical analysis

The primary endpoint was the incidence rate of new MACE over the follow-up period since hospital discharge after the primary ACS. MACE was defined as a composite of death by cardiovascular cause, non-fatal Myocardial Infarction (MI), non-fatal Cerebrovascular Accident (CVA), and repeat non-staged Percutaneous Coronary Intervention (PCI). Cardiovascular death was considered when the patient was admitted due to a MACE. This does not include patients admitted for non-cardiovascular events who subsequently developed ACS or CVA during their hospitalization. The non-fatal myocardial infarction was considered using the current universal definition of myocardial infarction at the time of admission [20].

Stroke was defined as compatible clinical symptoms plus imaging findings of brain injury on computed tomography or magnetic resonance imaging [21]. In our study, scheduled angioplasty was considered for all PCI procedures performed after one month from the primary event due to symptoms or ischemia on stress test.

The surveillance follow-up for MACE in each patient was conducted by the Primary Care Physician (PCP) or the Principal Cardiologist. Patients who were not followed up at our institution were contacted by phone by medical personnel who underwent a structured interview.

The secondary endpoint was the comparison of incidence rates of MACE in subpopulations of patients without prior radiotherapy in any body region, and in patients without active chemotherapy at the time of ACS. The objectives of secondary prevention were also derived from the Electronic Medical Record (EMR) data and were aligned with the current guidelines of the American College of Cardiology (ACC) and the European Society of Cardiology (ESC), conducted by the Primary Care Physician (PCP) or the Principal Cardiologist. These parameters were measured in both oncological and non-oncological patients as part of their follow-up.

We estimated a sample power for time-to-event analysis, considering an alpha and beta error of 0.05 and 0.2, respectively. Following the Friedman method, a total of 167 MACE over the follow-up were necessary to obtain a power of 80 %. Categorical variables are reported as a frequency and percentage and subgroups were compared using Chi-square or Fisher exact test, as appropriate. Continuous variables are reported as mean and standard deviation (±SD) or as median (IQR), and were compared using the *t*-test or Wilcoxon, for normal or non-parametric distribution, respectively. To determine whether cancer acted as an independent predictor of MACE, adjusted for traditional cardiovascular risk factors, a Cox regression model was conducted. Hazard ratios and their corresponding 95 %CI were estimated. Performance of the final model was evaluated through calibration (observed vs expected outcomes) and discrimination with Harrell's c-statistics index. Kaplan-Meier survival curves were computed and compared using the log-rank test. The same analysis was performed with the secondary objective subpopulations. All statistical analyses were performed with Stata® 17.0BE.

## 3 Results

**Fig. 1:**
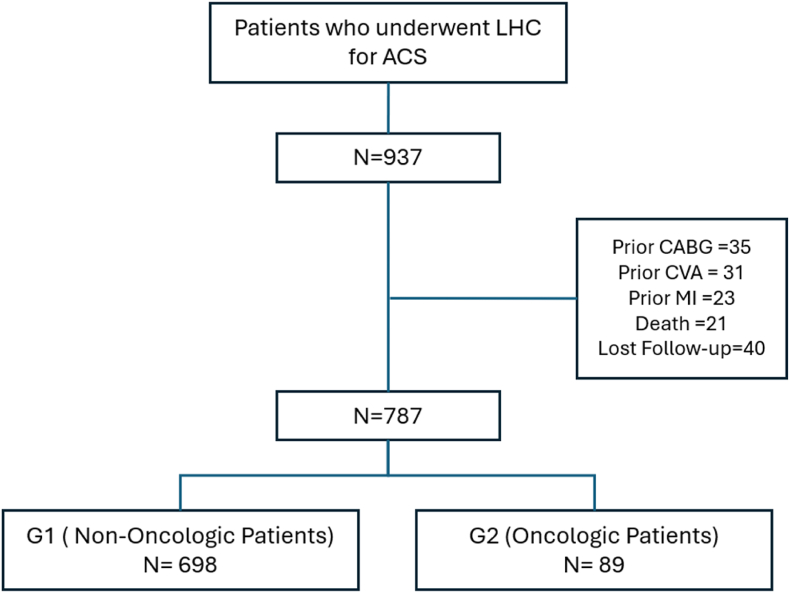
Flow chart of patient selection LHC (Left Heart Catheterization), ACS (Acute Coronary Syndrome), MI (Myocardial Infarction), CVA (Cerebrovascular disease), CABG (Coronary Artery Bypass Graft Surgery).

An initial screening process detected 937 patients who had been presented to our hospital for ACS and had undergone a LHC as part of their work-up. Of those patients, we excluded 35 patients who had undergone CABG, 31 CVA, prior myocardial infarction 23, death 21, and 40 patients were lost to follow-up. These patients who had been lost to follow-up were looked up on national registries to ensure they were not deceased. A total of 787 patients who completed follow-up were included, 698 (88.7 %) without cancer (G1) and 89 (11.3 %) with a history of cancer or active cancer (G2) (see Fig. 1). The median follow-up time for the entire cohort was 48 months (IQR = 15–72), being 48 months (IQR = 15–82) for G1 and 36 months (IQR = 12–48) for G2 (0.0004).

Patients with cancer were significantly older (67 years/±10 vs 60.2 years ± 11, p 0.0001) and had a higher prevalence of insulin-dependent diabetes mellitus (10 % vs 4 %, p = 0.01). The rest of the baseline characteristics can be seen in Table 1. The therapeutic strategies and general therapeutic objectives for the management of ACS were adjusted to the guidelines available at that time (Supplementary material 1). There were no significant differences in LHC findings between the two groups. The characteristics of the LHC can be seen in Table 1.

**Table 1:** Baseline Characteristics of both subpopulations at the time of ACS presentation.

Variables	Total (N = 787)	G1 (N = 698)	G2 (N = 89)	P Value
Age (years)	61 (SD ± 11)	60.2 (SD ± 11)	67 (SD ± 10)	<0.001
Male Sex	645 (82 %)	572 (81.9 %)	73 (82 %)	0.986
Hypertension	496 (63 %)	441 (63.2 %)	55 (61.8 %)	0.799
No DM	625 (79.4 %)	563 (80.5 %)	63 (71 %)	0.030
NIDDM	125 (17.5 %)	108 (15.5 %)	17 (19 %)	0.378
IDDM	37 (4.7 %)	28 (4 %)	9 (10 %)	0.010
History of Smoking	480 (61 %)	430 (62 %)	50 (56 %)	0.323
Dyslipidemia	494 (62.7 %)	442 (63.2 %)	52 (58.4 %)	0.368
Hyperuricemia	19 (2.41 %)	16 (2.29 %)	3 (3.7 %)	0.532
Obesity	290 (36.9 %)	267 (38.3 %)	23 (25.8 %)	0.022
Family History of Cardiovascular Disease	137 (17.4 %)	130 (18.6 %)	7 (7.9 %)	0.012
Sedentarism	441 (56 %)	405 (58 %)	36 (40.4 %)	0.002
Length of Stay (days)	3 (IQR 2–5)	3 (IQR 2–6)	4 (IQR 3–5)	0.151
Mean LVEF al time of discharge (%)	54 (SD ± 10.5)	54 (SD ± 10)	53 (SD ± 11)	0.445
**Number of Affected vessels**				
0	73 (9.27 %)	63 (9.02 %)	10 (11.2 %)	0.498
1	338 (42.9 %)	306 (43.8 %)	32 (35.9 %)	0.157
2	277 (35.2 %)	244 (34.9 %)	33 (37.1 %)	0.693
3	100 (12.7 %)	87 (12.5 %)	13 (14.6 %)	0.569
Left Main	20 (2.54 %)	18 (2.58 %)	2 (2.25 %)	0.851
Left Anterior Descending	475 (60.4 %)	421 (60.3 %)	54 (60.7 %)	0.948
Left Circumflex	314 (39.9 %)	276 (39.5 %)	38 (42.7 %)	0.567
Right Coronary	363 (46.1 %)	315 (45.1 %)	48 (53.9 %)	0.117
**Number of Treated Lesions**				
1	405 (51.4 %)	368 (53.5 %)	37 (41.6 %)	0.047
2	239 (30.4 %)	209 (30.3 %)	30 (33.7 %)	0.467
3	70 (8.89 %)	59 (8.6 %)	11 (12.3 %)	0.224

G1 = Non-Oncologic Group, G2 = Oncologic Group, DM = Diabetes Mellitus, NIDDM= Non-Insulin Dependent Diabetes Mellitus, IDDM= Insulin-Dependent Diabetes Mellitus.

The median time from cancer diagnosis to cohort entry was 24 months for G2. The cell line and staging characteristics of the oncology population can be seen in Supplementary material 2.

### 3.1 Incidence

A total of 173 (21.9 %) MACE were identified in the follow-up period in both groups combined. The cumulative incidence of MACE for G1 was 21.06 % (147/698) and for G2 was 29.3 % (26/89). G2 presented a significantly higher incidence density of MACE when compared to G1 [8 MACE/1000 patients/month (95 % CI 5.5–11.8) vs 4 MACE/1000 patients/month (95 % CI 3.4–4.8), p = 0.0032]. The rate of cardiovascular death during the follow-up period was 4.5 % for G2 and 0.14 % for G1 (P = 0.001). Other findings on the MACE events during the follow-up period can be found on Table 2.

**Table 2:** Incidence of major adverse cardiovascular events.

MACE	Total (N = 787)	G1 (N = 698)	G2 (N = 89)	P Value
**Total MACE**	173 (21.9 %)	147 (21.06 %)	26 (29.2 %)	0.001
**Cardiovascular Death**	5 (0.64 %)	1 (0.14 %)	4 (4.5 %)	0.001
**Nonfatal Myocardial Infarction**	101 (12.8 %)	90 (12.9 %)	11 (12.6 %)	0.132
**Nonfatal CVA**	4 (0.51 %)	2 (0.29 %)	2 (2.25 %)	0.009
**Repeat PCI**	63 (8 %)	54 (7.7 %)	9 (10.5 %)	0.056

∗P-value for incidence rate ratio.

∗∗ Causes of cardiovascular death in G2: three patients due to cardiogenic shock and one patient due to stroke (CVA).

The non-cardiovascular mortality of the entire cohort during the follow-up period was 1.01 %. In G2, non-cardiovascular death was higher than in G1 [5 (5.62 %) vs 3 (0.43 %), p = 0.001].

**Fig. 2:**
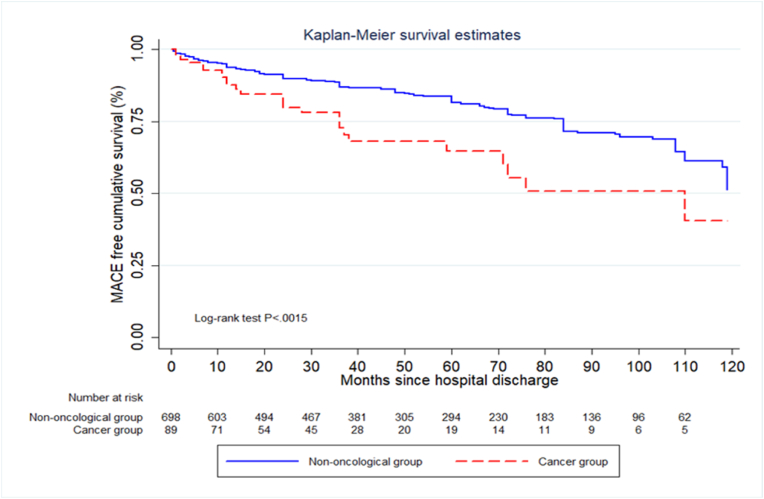
Kaplan Meier curve for time-to-MACE.

The Kaplan Meier curve showed a statistically significant difference in time-to-MACE between the two groups, with p = 0.0015 and a LR Chi 32.7 (See Fig. 2).

Multivariate Cox hazard analysis showed that cancer was an independent predictor of MACE (HR 1.84, [95 % CI 1.19–2.85] p = 0.006, Harrell's C p = 0.63), adjusted for hypertension, dyslipidemia, diabetes, history smoking, sedentary lifestyle, obesity, age, sex, and family history of cardiovascular disease (Table 3).

**Table 3:** Multivariate analysis for MACE.

Predictor	Hazard Ratio	95 % Confidence Interval	P Value
History of Cancer	1.84	1.2–2.84	0.006
Smoking history	1.21	0.86–1.69	0.258
Diabetes Mellitus	1.29	0.90–1.86	0.162
Age	1.01	0.99–1.02	0.230
Male Sex	1.36	0.87–2.12	0.171
Dyslipidemia	1.4	0.79–1.54	0.563
Obesity	0.88	0.63–1.23	0.462
Hypertension	1.68	1.17–2.40	0.005
Sedentarism	1.13	0.80–1.60	0.460
Family History	0.75	0.48–1.18	0.221

### 3.2 Secondary prevention quality

No differences were found in the use of DAPT or in the average statin doses. The LDL values of patients with MACE were similar between both groups. Table 4.

**Table 4:** Secondary Prevention goals during the follow-up period for MACE subgroups.

Secondary Prevention Profiles at the time of MACE	Total MACE population	G1 MACE (N = 147)	G2 MACE (N = 26)	P Value
Statin Usage	147 (84 %)	122 (83 %)	22 (84 %)	0.838
Mean Dose of Daily Atorvastatin (mg/dl)	40 (IQR = 20–40)	40 (IQR = 20–40)	40 (IQR = 20–40)	0.334
LDL (mg/dl)	79 (IQR = 61–101)	77 (IQR = 60–97)	84 (IQR = 76–107)	0.125
DAPT usage	148 (85.5 %)	123 (84.7 %)	22 (84 %)	0.904
Aspirin usage	148 (85.5 %)	125 (85 %)	23 (88 %)	0.647

∗DAPT: dual antiplatelet treatment, G1: no oncologic group, G2: Oncologic group Secondary Outcomes.

**Fig. 3:**
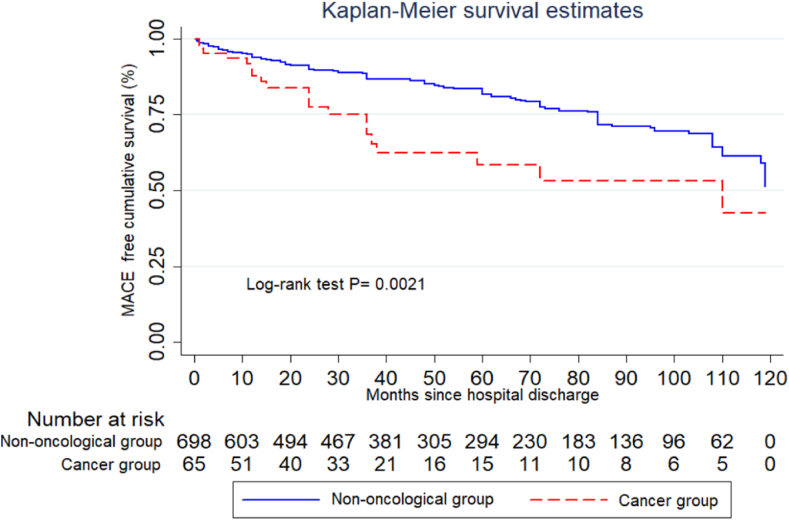
Kaplan-Meier Curve for Time to MACE comparing oncologic patients without prior Radiotherapy and non-oncologic patients.

A total of 65 patients with a history of cancer without prior radiotherapy were included in the subgroup analysis. The Cox analysis showed that Hazard Ratio (HR) of 1.92, (95 % CI 1.19–3.08) p = 0.007, adjusted for age, sex and the classic risk factors for the oncology population. The Kaplan Meier curve comparing patients without prior radiotherapy again demonstrated a statistically significant difference between the two groups, with a p of 0. 0021. Fig. 3.

**Fig. 4:**
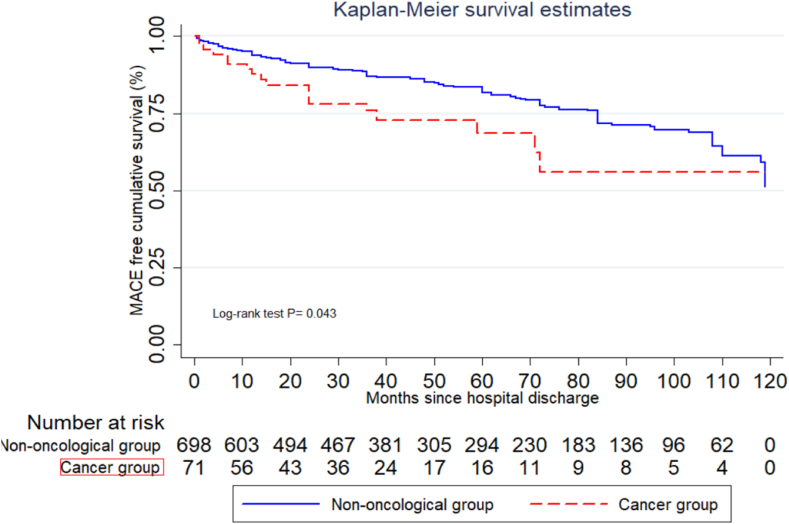
Kaplan-Meier Curve for Time to MACE comparing oncologic patients Without Active Chemotherapy and non-oncologic patients.

The subgroup of patients without active chemotherapy consisted of 71 patients. The total number of MACE in this subgroup analysis was 165, with 147 events in G1 and 18 events in G2. The Kaplan-Meier curve comparing both subgroups showed p = 0.043 (Fig. 4). Cox Hazard analysis showed a non-significant difference between the incidence of MACE between the two groups, with an HR 1.61 (95 % CI 0.97–2.68, p = 0.066), adjusted for the risk factors mentioned above (Supplementary material 3)

## 4 Discussion

Our study provides robust evidence that a history of cancer is an independent risk factor for major adverse cardiovascular events (MACE) in patients undergoing secondary prevention after a first episode of acute coronary syndrome (ACS). This finding holds significant clinical relevance, underscoring the importance of incorporating oncologic history into cardiovascular risk stratification and management frameworks. While previous research has demonstrated elevated cardiovascular risk in cancer patients—often attributed to malignancy-associated inflammation, endothelial dysfunction, and treatment-induced toxicity—the implications of cancer history within the context of secondary prevention remain underexplored.

In our cohort, a history of cancer was associated with an 84 % increased risk of MACE during follow-up. This excess risk was primarily driven by a higher incidence of cardiovascular death and stroke, whereas other MACE components did not reach statistical significance.

Importantly, the observed increase in cardiovascular mortality was not attributable to discrepancies in in-hospital management. Patients with a history of cancer received guideline-directed therapy, including timely primary or rescue percutaneous coronary intervention (PCI), comparable to those without cancer. In patients who underwent PCI, a complete revascularization strategy before discharge was pursued in cases of multivessel disease, with no statistically significant differences between groups. This consistent approach to coronary management likely contributed to the reduction in ischemic events and to the absence of differences in the incidence of non-fatal myocardial infarction between groups.

Another significant contributor to MACE was stroke. Although atrial fibrillation was not incorporated into the adjustment model, the Cox proportional hazards regression accounted for hypertension, diabetes mellitus, dyslipidemia, and age—each recognized as established risk factors for cerebrovascular events. Notably, the only two variables that remained statistically significant predictors of MACE in the Cox analysis were a history of cancer and hypertension.

Finally, it is worth noting that 75 % of cardiovascular deaths were secondary to myocardial infarction, rather than other cardiovascular causes such as heart failure or sudden arrhythmic death.

Left ventricular ejection fraction (LVEF) is a well-established prognostic marker following ACS [22,23]. In our study, LVEF at discharge did not differ significantly between groups, suggesting that reduced LVEF is unlikely to explain the observed association. Similarly, diabetes mellitus is a recognized independent risk factor for recurrent cardiovascular events [24,25]. However, MACE incidence among diabetic cancer patients (30 %) was comparable to diabetic patients without a cancer history (27 %), with no statistically significant difference, indicating that diabetes alone does not account for the increased cardiovascular risk in cancer patients.

Multiple mechanisms may underline the association between cancer and heightened MACE risk. Malignancy-related systemic inflammation, pro-thrombotic states, endothelial dysfunction, and metabolic derangements accelerate atherosclerotic progression and predispose to thrombosis. Additionally, oncologic therapies, including cytotoxic chemotherapy, targeted agents, and radiation therapy—have well-documented cardiotoxic profiles, which may exacerbate pre-existing coronary artery disease.

Radiation therapy has garnered attention as an emerging risk factor for coronary atherosclerosis [[26], [27], [28]]. To explore this, we conducted a subgroup analysis excluding patients with a history of radiotherapy. The association between cancer and MACE persisted, reinforcing the hypothesis that cancer history per se confers additional cardiovascular risk beyond the effects of radiation exposure.

Given increasing evidence of direct endothelial injury induced by oncologic therapies across age groups [9,[20], [29], [30]], we performed a second subgroup analysis excluding patients undergoing active chemotherapy. The association between cancer history and MACE remained statistically significant, further supporting the notion that cardiovascular risk persists beyond the active treatment phase.

Collectively, our findings support the concept that a history of cancer is associated with elevated MACE risk irrespective of the type or timing of oncologic therapy. Future studies are warranted to delineate the relative contributions of specific cancer types, therapeutic modalities, and latency periods to this risk.

Our results are consistent with those reported by Tabata et al. [16] and Wright et al. [31], who examined MACE incidence in cancer patients within a secondary prevention setting. However, unlike prior studies, our analysis controlled for treatment strategies and quality of secondary prevention. Despite differences in cohort characteristics, our findings align with theirs and emphasize the need for refined cardiovascular care in this population. Notably, our study population demonstrated >80 % adherence to secondary prevention targets, exceeding the benchmark reported in current guidelines [32].

The 2022 ESC Cardio-Oncology Guidelines [33] currently provide the most comprehensive framework for managing cardiovascular risk in cancer patients. Nonetheless, over 70 % of their recommendations are based on expert consensus (Level of Evidence C), and extrapolation from non-oncologic populations is common. In our cohort, no significant intergroup differences were found in DAPT duration, aspirin use at the time of recurrent events, or statin intensity. Although mean LDL levels did not meet current targets, they reflected the guideline thresholds in place at the cohort's inception (January 2008), which recommended <100 mg/dL for moderate-risk patients and <70 mg/dL for those with diabetes or high risk. Importantly, LDL levels did not differ significantly between patients who experienced MACE and those who did not.

On the other hand, no bleeding events were reported during follow-up, and the bleeding risk typically expected in oncologic patients was not observed. This favorable safety profile allowed for adherence to dual antiplatelet therapy (DAPT) of up to 80 % among patients who experienced MACE. A possible explanation for this finding is that, in the cancer group, the median time from cancer diagnosis to cohort inclusion was 26 months, and only 19 patients undergoing active cancer treatment met the inclusion criteria, which may have contributed to a lower bleeding risk than previously reported.

Emerging evidence highlights shared inflammatory mechanisms between atherosclerosis and cancer-related therapies, potentially explaining our findings [20,[34], [35], [36], [37]]. Further mechanistic studies are needed to validate this hypothesis.

Finally, our findings advocate for the expansion of cardio-oncology beyond primary prevention, to encompass secondary prevention strategies. A multidisciplinary approach, involving regular cardiovascular surveillance, individualized risk assessment, and optimization of evidence-based therapies, is essential to improving outcomes in this vulnerable patient population.

## 5 Limitations

Our study was designed as a retrospective observational study, which inherently limits data collection to what was available at the time of the study. This results in a lower level of evidence compared to prospective studies. Patients who did not undergo coronary angiography and were managed medically were excluded. This decision was based on the retrospective nature of the study and the evolution of the universal definition of myocardial infarction during the study period, to minimize misclassification—particularly considering the diagnostic complexity of ACS in patients with cancer. While including medically managed patients might have expanded the spectrum of clinical severity, it would have also introduced substantial diagnostic heterogeneity. Moreover, the relatively small number of oncology patients significantly limits the ability to assess the clinical impact of our findings, particularly in subgroup analyses and in the evaluation of specific cancer subtypes.

Another limitation is that while we adjusted for traditional cardiovascular risk factors, such as hypertension, smoking and diabetes, we did not account for other relevant comorbidities, including atrial fibrillation, frailty, low BMI, anemia, and autoimmune disorders, which have been associated with adverse cardiovascular outcomes [38]. Another important limitation is the inability to perform tumor subtype subgroup analyses due to the low number of patients. Additionally, due to insurance coverage restrictions, we were unable to assess the presence of clonal hematopoiesis of indeterminate potential (CHIP) within our cohort, despite its known association with hematologic malignancy and increased cardiovascular risk. Finally, secondary prevention goals, such as optimal LDL levels and statin dosages, varied throughout the cohort due to evolving clinical guidelines and emerging evidence.

## 6 Conclusion

In our population, cancer represents a potential novel and independent risk factor for MACE in patients undergoing secondary prevention following an ACS. Our findings emphasize the urgent need to incorporate oncologic history into cardiovascular risk models and management algorithms. Future research should focus on developing targeted preventive strategies and exploring potential therapeutic interventions to mitigate cardiovascular risk in this vulnerable population. A collaborative, multidisciplinary approach will be essential to improve long-term outcomes for patients with coexisting cancer and cardiovascular disease.

## CRediT authorship contribution statement

**Renzo Melchiori:** Writing – review & editing, Writing – original draft, Validation, Supervision, Project administration, Methodology, Investigation, Formal analysis, Conceptualization. **Sara Diaz Saravia:** Writing – original draft, Methodology, Data curation. **Pablo M. Rubio:** Writing – original draft, Investigation, Data curation. **Lucas Szlaien:** Writing – original draft, Data curation. **Romina Mouriño:** Writing – original draft, Investigation, Data curation. **Martin O'Flaherty:** Writing – review & editing, Validation. **Manglio Rizzo:** Writing – review & editing, Validation, Supervision, Methodology, Conceptualization. **Alejandro Hita:** Writing – review & editing, Validation, Supervision, Methodology, Conceptualization.

## Disclosures

This manuscript received no financial support. The authors have no conflicts of interest. All authors listed contributed equally to the development of this study.
