# Supplementary material for: Cancer as a novel risk factor for major cardiovascular adverse events in secondary prevention
Source: Int J Cardiol Cardiovasc Risk Prev. 2025 Aug 26;27:200501. doi: 10.1016/j.ijcrp.2025.200501 (PMC12445231; doi:10.1016/j.ijcrp.2025.200501)
Supplement: Multimedia component 1 [file mmc1.docx]

Supplementary data

Table S 1: Therapeutic Strategies of ACS.

| **Therapeutic Strategy of ACS** | **Total 787** | **G1 698** | **G2 89** | **P Value** |
| --- | --- | --- | --- | --- |
| Medical Treatment  Fibrinolytics  Angioplasty  CABG | 73 (9.27%)  1 (0.13%)  670 (85.5%)  43 (5.64%) | 63 (9%)  1 (0.14%)  597 (86.5%)  37 (5.29%) | 10(11.23%)  –  73 (82.65%)  6 (8.16%) | 0.498  -  0.381  0.573 |

**Table S2: Bleeding complications related to the procedure. BARC scale (Bleeding Academic Research Consortium).**

| **BARC Scale** | | | | |
| --- | --- | --- | --- | --- |
|  | **Total N:787** | **G1 N: 698** | **G2 N: 89** | **P value** |
| **0** | 579 (73.6%) | 478 (68.4%) | 67 (75.3%) | 0.190 |
| **1** | 188 (23.9%) | 202 (28.9%) | 20 (22.5%) | 0.202 |
| **2** | 16 (2.03%) | 15 (2.15%) | 1 (1.12%) | 0.443 |
| **3** | 4 (0.51%) | 3 (0.43%) | 1 (1.12%) | 0.382 |

Table S3:Oncologic Population Cell line and staging of malignancies.

| **Variable** | **Solid Malignancies (N=73)** | | **Heme Malignancies (N=16)** | |
| --- | --- | --- | --- | --- |
| **Cell Line/Location** | Prostate | 16 (22%) | Lymphoma | 8 (50%) |
|  | Colon | 12 (16.4%) | Multiple Myeloma | 2 (12.5%) |
|  | Kidney | 7 (9.6%) | CML | 2 (12.5%) |
|  | Bladder | 7 (9.6%) | CLL | 2 (12.5%) |
|  | Lung | 5 (6.8%) | MDS | 2 (12.5%) |
|  | Breast | 5 (6.8%) |  |  |
|  | Rectum | 4 (5.5%) |  |  |
|  | Larynx | 3 (4.1%) |  |  |
|  | Pancreas | 3 (4.1%) |  |  |
|  | Melanoma | 2 (2.7%) |  |  |
|  | Other | 9 (12.3%) |  |  |
| **Stage I** | 23 (31.5%) | | 3 (18.8%) | |
| **Stage II** | 14 (19.2%) | | 6 (37.5%) | |
| **Stage III** | 13 (17.8%) | | 3 (18.8%) | |
| **Stage IV** | 17 (23.3%) | | 3 (18.8%) | |
| **Unknown** | 6 (8.2%) | | 1 (6.3%) | |

Table S4 Other Characteristics of the oncologic group.

| **Variable** | **Solid Malignancies (N=73)** | **Heme Malignancies (N=16)** |
| --- | --- | --- |
| **Median Time from Dx to ACS** | 26 months (IQR 8-36) | 22 months (IQR 8-34) |
| **Prior Radiotherapy** | 23 (31%) | 1 (6.3%) |
| **Active Chemotherapy** | 15 (20.5%) | 4 (25%) |
| **Prior Immunotherapy** | 4 (5.5%) | 8 (50%) |
